# Assessing the Impact and Cost of Short-Term Health Workforce in Remote Indigenous Communities in Australia: A Mixed Methods Study Protocol

**DOI:** 10.2196/resprot.5831

**Published:** 2016-10-03

**Authors:** John Wakerman, John Humphreys, Lisa Bourke, Terry Dunbar, Michael Jones, Timothy A Carey, Steven Guthridge, Deborah Russell, David Lyle, Yuejen Zhao, Lorna Murakami-Gold

**Affiliations:** ^1^ Flinders NT School of Medicine Flinders University Darwin Australia; ^2^ Monash University School of Rural Health Monash University Bendigo Australia; ^3^ University Department of Rural Health The University of Melbourne Shepparton Australia; ^4^ Yaitya Purruna Indigenous Health Unit University of Adelaide Adelaide Australia; ^5^ Psychology Department Macquarie University Sydney Australia; ^6^ Centre for Remote Health Flinders University and Charles Darwin University Alice Springs Australia; ^7^ Northern Territory Department of Health Darwin Australia; ^8^ Broken Hill University Department of Rural Health The University of Sydney Broken Hill Australia

**Keywords:** remote health, rural workforce, fly-in/fly-out, rural health services, health services, Indigenous

## Abstract

**Background:**

Remote Australia is a complex environment characterized by workforce shortages, isolated practice, a large resident Indigenous population, high levels of health need, and limited access to services. In recent years, there has been an increasing trend of utilizing a short-term visiting (fly-in/fly-out) health workforce in many remote areas. However, there is a dearth of evidence relating to the impact of this transitory workforce on the existing resident workforce, consumer satisfaction, and the effectiveness of current services.

**Objective:**

This study aims to provide rigorous empirical data by addressing the following objectives: (1) to identify the impact of short-term health staff on the workload, professional satisfaction, and retention of resident health teams in remote areas; (2) to identify the impact of short-term health staff on the quality, safety, and continuity of patient care; and (3) to identify the impact of short-term health staff on service cost and effectiveness.

**Methods:**

Mixed methods will be used. Administrative data will be extracted that relates to all 54 remote clinics managed by the Northern Territory Department of Health, covering a population of 35,800. The study period will be 2010 to 2014. All 18 Aboriginal Community-Controlled Health Services in the Northern Territory will also be invited to participate. We will use these quantitative data to describe staffing stability and turnover in these communities, and then utilize multiple regression analyses to determine associations between the key independent variables of interest (resident staff turnover, stability or median survival, and socioeconomic status, community size, and per capita funding) and dependent variables related to patient care, service cost, quality, and effectiveness. The qualitative component of the study will involve in-depth interviews and focus groups with staff and patients, respectively, in six remote communities. Three communities will be high staff turnover communities and three characterized by low turnover. This will provide information on service quality, impact on resident and visiting staff, and patient satisfaction with the services. The research team will work with staff, patients, and a key stakeholder group of senior policymakers to develop workforce strategies to maintain or attain remote health workforce stability.

**Results:**

The study commenced in 2015. As of October 2016, fieldwork has been almost completed and quantitative analysis has commenced. Results are expected to be published in 2017.

**Conclusions:**

The study has commenced, but it is too early to provide results or conclusions.

## Introduction

In Australia, mortality rates increase with increasing distance from major cities [1]. Access to health services declines with increasing remoteness; consequently, rates of preventable admissions to hospital increase markedly with increasing remoteness [2]. Therefore, nowhere is it more urgent to ensure the strongest possible primary health care system than in remote areas, including in Indigenous communities, in order to prevent illness, serious complications, and the avoidable expense of hospitalization.

There has always been a need for some visiting services to small, remote settlements where population size does not support a full range of resident primary and specialist services [3]. More recently, there has been an increasing reliance on short-term or “fly-in/fly-out” (FIFO) or “drive-in/drive-out” (DIDO) services to overcome the health workforce recruitment and distribution problems in remote Australia, and a concomitant proliferation of private staffing agencies contributing to this workforce trend [4]. Increasing use of short-term or agency staff, who move from place to place or are one-off visitors, has raised significant concerns about the impact on patients and resident health service staff [5].

The provision of primary health care by nonresident staff in remote areas of Australia is characterized by different forms of visiting services [3]:

1. Specialist medical outreach services;

2. Hub and spoke or outreach arrangements for various allied health and specialized programs, such as women’s health educators or mobile dental services;

3. “Orbiting staff” who spend significant periods of time (12 months or more) in one or two specific communities, self-regulate stress levels, and work elsewhere for periods then return to the same communities where orientation is not required;

4. Long-term shared positions, such as month-on/month-off, where the same practitioners service the same communities;

5. Experienced locum relief for resident staff; and

6. FIFO/DIDO or short-term or agency staff who move from place to place.

Although the need for short-term relieving or locum staff is legitimate, expedient, and should be met, there are associated risks that may increase in situations where the resident team is partially or largely replaced by short-term staff. The limited available evidence suggests that these risks include increased stress on resident staff, increased costs, decreased effectiveness of services resulting in increased hospital admissions, and suboptimal coordination of services. The House of Representatives Standing Committee on Regional Australia has expressed concern “that a FIFO heath workforce will undermine a residential health workforce and lead to the closure of existing facilities” (p 151 [5]).

The high turnover associated with short-term staff results in existing staff members repeatedly orienting new staff [6], which in turn results in additional pressure on long-term staff who become more stressed [7]. The “emerging lack of parity in their employment terms and conditions granted to the FIFO and DIDO workforce” also makes retaining existing long-term staff more difficult (p 17 [8]).

Over time, resident remote area staff develop a detailed knowledge of their communities and those communities’ health needs. A resident registered nurse or midwife is more engaged with the local community and better placed to function effectively in a remote setting than visiting teams [9]. The effectiveness of primary health care services is predicated on strong relationships, good communication, and trust, especially in Aboriginal communities [10].

Short-term staff may not have the knowledge and experience necessary for clinical work in remote Australia [4,6,11]. Cultural competence is important when providing health services to Aboriginal communities [10], and there are concerns about a lack of preparation for remote practice of short-term staff [8].

Effective health care in remote settings requires the coordinated implementation of health care plans involving different health professionals. However, it is difficult to both coordinate multiple visiting services and effectively implement these plans with a preponderance of short-term staff [6]. This can result in the constant “bombardment” of communities, which have neither accommodation nor resident staff capacity to support visiting professionals or to allow for necessary skills development [10]. An absence of stable and strong resident remote primary health care teams risks the “hollowing out” of these communities [5].

International evidence in relation to outreach (visiting) services is scant [12]. In Australia, there is “a dearth of empirical evidence” relating to the increasing trend of a short-term, visiting health workforce in remote areas, and its impact on the existing workforce and the effectiveness of current services [5]. The recent parliamentary enquiry recommended further research into the economic impact and the service impact of short-term FIFO staff in order to inform an appropriate health policy response. This is a pressing national health workforce issue that, to date, has not been informed by comprehensive, rigorous, and reliable research evidence.

Given these concerns about the potential adverse effects of FIFO/DIDO remote health staffing, the aim of the study described in this paper is to gather rigorous evidence of the extent to which a high level of short-term staffing in remote communities influences service acceptability to patients and the impact on permanent resident primary health care staff, service effectiveness, and cost. The specific objectives of the study are:

1. To identify the impact of short-term health staff on the workload, professional satisfaction, and retention of resident health teams in remote areas;

2. To identify the impact of short-term health staff on the quality, safety, and continuity of patient care; and

3. To identify the impact of short-term health staff on service cost and effectiveness.

## Methods

The study is underpinned by a logic model that links health service inputs (workforce), outputs, and outcomes. A mixed methods approach will capture the best-available quantitative data and in-depth primary data collected from stakeholder interviews and focus groups. A mixed methods approach is necessary because (1) this is a complex health system issue that includes service delivery in an equally complex, remote, cross-cultural context; (2) some measures are quantitative by nature (eg, staff turnover rate) and others are qualitative (eg, patient experience); and (3) some quantitative measures are likely to require explanatory qualitative data to be thoroughly understood. The quantitative and qualitative components of the study are described separately subsequently, followed by how they are integrated to address each study objective. The study has been approved by the Central Australian Human Research Ethics Committee (HREC-15-296) and the Human Research Ethics Committee of the Northern Territory Department of Health and Menzies School of Health Research (2015-2363).

### Setting and Participants:

The study sites include all 54 remote clinics managed by the Northern Territory (NT) Department of Health, covering a population of 35,800. The study period will be 2010 to 2014. This period will generate data related to approximately 480 full-time equivalent staff, 1,621,000 primary health care visits, and 271,000 hospital admissions. In addition, we have support for the project from the peak body for Aboriginal Community-Controlled Health Services, the Aboriginal Medical Services Alliance of the Northern Territory (AMSANT), and we will individually invite 18 remote community-controlled health services to also participate.

### Measures

#### Measures of Staff Stability

The extent of utilization of short-term primary health care workers will be determined by calculating:

1. Annual resident primary health care workforce turnover (this includes resident doctors, nurses, and Aboriginal Health Practitioners) as measured by (number of leavers per year × 100)/average number employed per year;

2. Workforce stability as measured by (number of original entrants surviving at the end of each year × 100)/number of original entrants; and

3. Median survival of staff members.

These will be the major measures of inputs as per the underpinning logic model.

#### Quality and Cost-Effectiveness Outcomes

These measures will be used to evaluate quality of health care provision (objective 2) and cost-effectiveness (objective 3). The outputs are (1) expenditure by clinic and per capita (relates to objective 3) and (2) utilization as measured by attendances per clinic (relates to objective 2).

Quality indicators include (1) the proportion of diabetics with a chronic disease management plan, (2) proportion of eligible adults with an annual Adult Health Check, (3) proportion of diabetics with proteinuria on appropriate renal protective medication, (4) proportion of patients with cardiac disease on acetylsalicylic acid, (5) timely antenatal care, (6) Pap smear coverage, (7) immunization coverage, and (8) proportion of children screened for anemia (objective 2).

Intermediate outcomes include (1) numbers of medical retrievals by clinic (objective 2) and (2) preventable admissions to hospital by clinic (objective 2). Clinical outcomes include (1) proportion of known diabetics with blood sugar controlled (HbA_1c_<7%) (objective 3), (2) proportion of known hypertensives with controlled blood pressure (objective 3), and (3) mortality estimates by location (objective 3).

A number of potential confounding variables that may contribute to dependent variables of interest will be considered in the regression model. They include (1) measures of socioeconomic status, (2) variability of funding and staffing between clinics, and (3) size of communities (related to economies of scale).

Several additional factors may potentially limit the analysis. These include:

1. Patient migration. With declining health, there is a small movement of people to larger centers [13]. We know that there is approximately 90% accuracy in identifying place of residence [14].

2. The relationship of primary health care utilization and hospital admission is not linear [15].

3. Specialist and allied health outreach visits. The effectiveness of these services is affected in a similar fashion by high staff turnover.

4. Quality of governance.

5. Intergenerational changes in attitude toward employment.

### Data Sources and Feasibility

This study uses NT Government administrative datasets, including hospital admissions, primary health care visits, patient travel, government payroll, and accounting system. A remote health administrative roster and outreach diaries are also available for analysis. The NT Aboriginal Health Key Performance Indicators, including all quality measures, are routinely collected by both government and nongovernment health services. Data are comprehensive and reliable. Given appropriate ethics and data custodian approvals, data are available and accessible at a deidentified individual level, such as diagnosis codes for patients, position classifications for health staff, employee start and end dates, and personnel and operational expenses. All these data have been investigated previously by members of the research team in other studies. Quality, completeness, and accuracy of the data are acceptable for this project. All these data have been collected consistently throughout the study period.

### Statistical Analysis

The quantitative component of the study has three elements that will be addressed as follows.

#### Description of Longevity of Clinic Staffing

The statistical approach to the first element will be addressed using descriptive statistics for quantitative measures of staff turnover and stability for the period from 2010 to 2014 (see Measures of Staff Stability). Because staff commencement and departure dates are recorded, survival methods will also be used to describe staff loss as a function of time, which allows for the possibility of right-censoring for staff who have not left at the time of study end.

#### Association Between Staff Stability and Outcomes

The second element of study design will utilize multiple regression analyses to determine associations between the key independent variables of interest (resident staff turnover, stability or median survival and socioeconomic status, community size, and per capita funding) and the dependent variables related to patient care, service cost, quality, and effectiveness.

#### Moderation of the Association Between Staff Stability and Outcomes

The third element will test whether socioeconomic status, community size, and per capita funding have an effect on the relationship between staff turnover on the dependent variables of interest via generalized linear models. That is, is the effect of staff turnover dependent or independent of socioeconomic status, community size, and per capita funding?

For both the second and third elements, formal statistical inference (hypothesis testing) will employ the nonparametric bootstrap method due to the expected nonnormal distribution of the quantitative dependent variables.

Analyses will be stratified to compare between government and nongovernment services, as well as by age groups (to examine intergenerational differences) and gender.

### Sample Size

Based on a minimum practically important effect size of a partial *r*^2^ of 5% when controlling for potentially confounding variables that explain 10% of the variance, an effective sample size of 260 patient records is required to achieve statistical power of .9 at the .01 (two-tailed) level of statistical significance. Because patients will be effectively cluster sampled from the participating clinics and substantial within-clinic correlation is expected, we assume a Kish design effect of 2.0, leading to a recruitment aim of 520 patients that can be easily achieved.

### Qualitative Methods

#### Study Sites and Participants

To assess patients’ and remote health professionals’ experiences of FIFO health care, to provide contextual information to the previously described statistical analyses, and to confirm the contribution of FIFO/DIDO to workforce turnover rates, six study sites will be examined using qualitative methods. Initial quantitative assessment of resident workforce turnover, stability, and median length of stay will differentiate between high and low staff turnover communities (stage 1). In stage 2, clinics from the upper and lower quartiles of turnover in NT will be invited to participate until three sites at either end of the turnover range (high and low) agree to participate.

In each of the six consenting study sites, two local community-based coresearchers (hereafter referred to as “coresearchers”), one male and one female, will be employed to work as part of the research team. The process of recruitment of female and male coresearchers at each study site will depend on the recommendations from community Elders, leaders, and key organizations to ensure the male and female coresearchers will be able to work together and work with multiple families in the community. Selection of suitable coresearchers is a critical and important process because this complex and multifaceted role will provide a cultural and linguistic interface with community members in each consenting study site. The coresearchers will assist with participant recruitment, organization, group facilitation, ensuring the research protocol is adapted to local cultural protocols and practicalities, interpreting, and back-translation for those participants for whom English is not their first language. The role is not restricted to these activities, but is adapted to ensure the research is culturally appropriate and the information gained is genuine. Individualized training (including a full explanation of the project, ethical processes to recruit participants, and the conduct of focus groups with Aboriginal community members) will be delivered for the coresearchers and tailored to their existing experience, skills identified, needs, and aspirations. The aim is to collect qualitative data from health professionals as well as patients.

First, after written consent is provided, researchers will conduct semistructured, face-to-face individual interviews with resident health professionals [16]. Individual interviews allow for confidentiality when perspectives may be diverse. For cultural safety reasons related to power differentials, the coresearchers may not wish to undertake individual interviews with resident health professionals. These interviews will explore resident staff experiences of short-term periodic staff, specifically issues of effectiveness of service delivery, motivation to work and remain in remote areas, job satisfaction, workload and stress, community engagement, and possible strategies to stabilize the workforce. Short-term staff will also be interviewed about their work, integration into the team, relationships with the community, and their perspective of effective service delivery. All interviewees will have the opportunity to raise issues about health care that they believe are important.

Second, coresearchers will recruit patients to participate in either semistructured, face-to-face individual interviews or focus groups, as appropriate. The coresearchers will cofacilitate the focus groups and either interview or identify the appropriate interviewer from the team [17]. Coresearchers with team members will discuss and come to agreement with community participants which method is culturally safe and preferred, considering issues of cultural safety, confidentiality, use of services, and preference of individuals. Focus groups tend to be more culturally appropriate and allow for a community rather than an individual narrative. At the same time, there may be community or individual issues best not discussed in an open forum. We estimate four interviews with patients as well as four focus groups (led by coresearchers and supported by another team member) will be conducted in each community, with approximately 10 participants in each group. Focus groups will be gender specific and respect age differences and clan differences. These group discussions will explore health service issues, including acceptability, experiences with staff and services, relationships with health service staff, and managing health issues that require sensitivity in relation to cultural issues. Although there will be guiding discussion points for the focus groups and the semistructured interviews, participants will have the opportunity to “tell their story” and express related information around the personal, family, and community impacts of short-term and high turnover of staff in the clinics. The focus will be on accurately recording these “stories” by taking notes, taping, and back-translating. All interviews and focus groups either will be audio recorded or have notes taken depending on participants’ consent. Coresearchers will also be encouraged to reflect on their experiences as researchers, through written or oral recordings, to contribute to understanding the research process, the context of the research, findings, and their summary of the findings.

Third, regional center-based specialist and retrieval staff will also be interviewed to assess quality of remote area services and specific issues such as medical evacuations from remote communities.

### Analysis

Interview and focus group recordings will be transcribed and analyzed with the assistance of NVivo. The patient focus group, patient interview, and health professional interview data will be analyzed separately because the transcripts are derived from different methods with different types of respondents (ie, they are different datasets). To begin, three researchers will read all transcripts to identify relevant issues. Community-based coresearchers will be asked to read transcripts or listen to audio recordings from their own community. Three researchers and the coresearchers will together identify codes and the three researchers will independently code each dataset. The three researchers and coresearchers will then agree on major themes for each dataset that blend codes and include local knowledge. These themes aim to describe the issues, underpinnings, and contexts that explain health care in these study sites [18]. Following, narrative analysis will be conducted to capture the stories of how the FIFO workforce has or has not shaped health and health care in these communities focusing on the patient stories [19]. Sampling high and low turnover clinics will allow comparison of service and contextual issues that accelerate or impede turnover of staff. There will also be comparison of findings from government and Aboriginal Community-Controlled Health Services.

#### Integration of Quantitative and Qualitative Information

Quantitative and qualitative data will be triangulated to address the three study objectives as follows:

1. The impact of visiting short-term health staff on resident health teams in remote areas will be measured by (stage 1) analysis of remote staff turnover, stability, and median survival to determine high and low turnover communities [20] and (stage 2) in-depth interviews with long-term staff, including Aboriginal Health Practitioners, remote area nurses, and medical officers to determine impact on staff work life (eg, morale, workload, stress, and intention to stay). Interviews with short-term staff will document similar issues as well as preparation for remote areas, work satisfaction, and level of community engagement.

2. Impact on the quality, safety, and continuity of patient care will be assessed through quantitative analysis of service quality data (as specified subsequently), in-depth interviews and focus groups with patients about their experience of the impact of short-term staff and their satisfaction with and acceptability of services, and interviews with specialist and retrieval staff about the quality of remote consultations and perceived need for medical evacuations.

3. Impact on service cost and effectiveness will be assessed by an analysis of expenditure, utilization, medical retrieval, and clinical outcome data in remote clinics for each community.

Triangulation of quantitative and qualitative data will assess whether the influence of high short-term staff utilization on objective markers of quality, cost, and effectiveness of health care services is paralleled in staff satisfaction, patient satisfaction, and service acceptability.

### Knowledge Exchange

Knowledge exchange is an integrated feature of the project. The research team has had extensive experience in research translation and has published on the measurement of research impact [21]. The knowledge exchange strategy will be multifaceted and include in-depth interviews with staff and patients to determine current and potential strategies to maintain or achieve remote health workforce stability; feedback to the study sites through the researchers; establishment of a key stakeholder group; presentations to national conferences; presentations to smaller forums, such as invited seminars to Commonwealth Department of Health staff; meetings with senior policymakers at Federal and State levels; and peer-reviewed publications. The key stakeholder group will comprise senior policy makers from NT Department of Health, Top End and Central Australian Health Services, NT Primary Health Network, AMSANT, the National Rural Health Alliance, and the Commonwealth Department of Health. Working with the key stakeholder group, workforce strategies will be developed based on research findings through “collaborative problem solving between researchers and decision makers that happens through...interaction between decision makers and researchers and results in mutual learning through the process of planning, producing, disseminating, and applying existing or new research in decision making” (p 15 [22]).

## Discussion

This study aims to build the currently deficient evidence base relating to a complex, real-world health systems issue: the impact of short-term staffing on the quality and costs of remote primary health care services. The study involves working in an equally complex remote, cross-cultural setting, involving multiple primary health care providers. It is a challenging real-world problem that requires a comprehensive, mixed methods approach to understand both the “what” and “why.” The direct involvement of health services, local researchers, a high-level key stakeholder group, and a comprehensive knowledge exchange strategy will help generate solutions and maximize the impact of the results on policy and practice.

## Abbreviations

AMSANT: Aboriginal Medical Services Alliance of the Northern Territory
DIDO: drive-in/drive-out
FIFO: fly-in/fly-out
NT: Northern Territory

## References

[1] (2005). Rural, Regional and Remote Health: Indicators of Health. Rural Health Series no 5. Cat. No. PHE 59. Australian Institute of Health and Welfare.

[2] COAG Reform Council (2011). Healthcare 2011-12: Comparing Performance Across Australia.

[3] Wakerman J, Curry R, McEldowney R (2012). Fly in/fly out health services: the panacea or the problem?. Rur Remote Health.

[4] Allen and Clarke (2011). Evaluation of the Child Health Check Initiative and the Expanding Health Service Delivery Initiative: Summary Report.

[5] House of Representatives Standing Committee on Regional Australia (2013). Cancer of the Bush or Salvation for our Cities?: Fly-in, Fly-out and drive-in, drive-out workforce practices in Regional Australia.

[6] Guerin P, Guerin B (2009). Social effects of fly-in-fly-out and drive-in-drive-out services for remote indigenous communities. Aust Commun Psychol.

[7] Lenthall S, Wakerman J, Opie T, Dollard M, Dunn S, Knight S, Macleod M, Watson C (2009). What stresses remote area nurses? Current knowledge and future action. Aust J Rural Health.

[8] Health Workforce Australia (2013). National Rural and Remote Workforce Innovation and Reform Strategy.

[9] Birks M, Mills J, Francis K, Coyle M, Davis J, Jones J (2010). Models of health service delivery in remote or isolated areas of Queensland: a multiple case study. Aust J Adv Nurs.

[10] Battye K, McTaggart K (2003). Development of a model for sustainable delivery of outreach allied health services to remote north-west Queensland, Australia. Rural Remote Health.

[11] Allen O (1996). Anthill and other injuries: a case for mobile allied health teams to remote Australia. Aust J Rural Health.

[12] De Roodenbeke E, Lucus S, Rouzaut A, Bana E (2011). Outreach Services as a Strategy to Increase Access to Health Workers in Remote and Rural Areas.

[13] Zhao Y, Condon J, Li SQ, Guthridge SL, Chondur R (2013). Indigenous patient migration patterns after hospitalisation and the potential impacts on mortality estimates. Australas J of Regional Stud.

[14] Foley M, Zhao Y, Condon J (2012). Demographic Data Quality Assessment for Northern Territory Public Hospitals.

[15] Zhao Y, Wright J, Guthridge S, Lawton P (2013). The relationship between number of primary health care visits and hospitalisations: evidence from linked clinic and hospital data for remote Indigenous Australians. BMC Health Serv Res.

[16] Minichiello V, Aroni R, Hays TN (2008). In-Depth Interviewing: Principles, Techniques, Analysis.

[17] Krueger R, Casey MA (2008). Focus Groups: A Practical Guide for Applied Research.

[18] Miles M, Huberman AM (1994). Qualitative Data Analysis: An Expanded Sourcebook.

[19] Greenhalgh T, Hurwitz B, Skultans V (2004). Narrative Research in Health and Illness.

[20] Russell DJ, Humphreys JS, Wakerman J (2012). How best to measure health workforce turnover and retention: five key metrics. Aust Health Rev.

[21] Buykx P, Humphreys J, Wakerman J, Perkins D, Lyle D, McGrail M, Kinsman L (2012). 'Making evidence count': a framework to monitor the impact of health services research. Aust J Rural Health.

[22] Graham ID, Logan J, Harrison MB, Straus SE, Tetroe J, Caswell W, Robinson N (2006). Lost in knowledge translation: time for a map?. J Contin Educ Health Prof.

